# The Effects of 40 Hz Gamma Binaural Beat Auditory Stimulation on Postural Control, Motor Coordination, and Cognitive Function Among Healthy Adults: A Pilot Study

**DOI:** 10.3390/jcm15187041

**Published:** 2026-09-11

**Authors:** Rehab Alhasani, Lujain Almutairi, Reyof Alshahrani, Seham Almutairi, Amal Alharbi, Remas Aldamok, Monira I. Aldhahi

**Affiliations:** Department of Rehabilitation Sciences, College of Health and Rehabilitation Sciences, Princess Nourah bint Abdulrahman University, P.O. Box 84428, Riyadh 11671, Saudi Arabia; rsalhasani@pnu.edu.sa (R.A.); 442001159@pnu.edu.sa (L.A.); 442004234@pnu.edu.sa (R.A.); 441000274@pnu.edu.sa (S.A.); 442000683@pnu.edu.sa (A.A.); 442004742@pnu.edu.sa (R.A.)

**Keywords:** binaural beats, gamma auditory stimulation, postural control, motor coordination, cognitive function

## Abstract

**Background/Objectives:** Auditory sensory stimulation, particularly binaural beats, affects neural oscillations and may improve cognitive and motor functions. Nevertheless, there is a paucity of research examining the concurrent effects of 40 Hz gamma binaural beats on postural control, motor coordination, and cognitive function in healthy adults. To evaluate the effects of 40 Hz gamma binaural beat auditory stimulation on postural control, motor coordination, and cognitive function in healthy adults. **Methods:** A pre–post quasi-experimental study was conducted among healthy adults who received 20 min of daily 40 Hz gamma binaural beat stimulation for two weeks. Outcomes included static balance, dynamic balance, upper-limb coordination, and cognitive function. **Results:** Thirty-two participants (mean age: 22.28 ± 2.43 years; 96.9% female) completed the study. Significant improvements were observed in dynamic balance (*p* < 0.001; d = 0.85), upper limb coordination (*p* < 0.001; d = 0.92), and PROMIS cognitive function scores (*p* < 0.001; d = 0.96). Static balance outcomes showed limited changes, except for the eyes-open Stability Index (*p* = 0.0005; d = −0.68). **Conclusions:** In this single-arm exploratory pilot study, 40 Hz gamma binaural beat stimulation was associated with improvements in selected sensorimotor and cognitive outcomes; however, in the absence of a sham or acoustically matched control condition, these findings should be interpreted as hypothesis-generating effect-size estimates rather than evidence of stimulation-specific efficacy, pending confirmation in controlled trials.

## 1. Introduction

Auditory stimulation has emerged as a promising non-invasive approach for modulating neural activity through brainwave entrainment mechanisms [1]. Among the various forms of auditory stimulation, binaural beats have attracted increasing scientific interest because of their potential to synchronize endogenous neural oscillations with externally delivered rhythmic stimuli [1,2]. Through this process, auditory stimulation may influence not only cognitive and emotional processes but also sensorimotor functions, including postural control and motor coordination [1,3].

Gamma binaural beats (GBB) are generated when two pure tones of slightly different frequencies are presented separately to each ear, creating the perception of a third frequency corresponding to the difference between the two tones [2]. This auditory phenomenon is believed to induce neural entrainment by synchronizing brain activity with the perceived frequency of beats. The interaction of these acoustic signals is thought to occur within the brainstem and auditory cortex, producing a phantom binaural beat that can influence cortical oscillatory activity [2,4].

Neural oscillations play a fundamental role in sensory processing, motor control, and cognitive performance. Among the electroencephalographic frequency bands, gamma oscillations are particularly associated with sensory integration, attention, working memory, memory encoding and retrieval, and higher-order cognitive processing [5]. Gamma-band activity has also been implicated in motor preparation, movement execution, and sensorimotor integration, highlighting its potential relevance to both cognitive and motor performance [5]. Consequently, modulating gamma activity through auditory entrainment may enhance functional performance across multiple domains.

The 40 Hz gamma frequency has received particular attention because of its strong association with cognitive processing and neural synchronization [6,7]. Experimental evidence suggests that 40 Hz stimulation may promote cortical coherence and facilitate communication between distributed neural networks [1,8]. Supporting this hypothesis, Wang et al. demonstrated that 40 Hz binaural beat stimulation improved working memory performance in healthy adults and was associated with neurophysiological changes indicative of enhanced neural entrainment and attentional processing [9]. Furthermore, 40 Hz stimulation has been shown to elicit strong neural responses within the prefrontal cortical regions and appears to be the preferred stimulation frequency for auditory cortical circuits [1,8].

Postural control depends on the coordinated integration of visual, vestibular, and somatosensory information to maintain stability and regulate movement. Previous studies have demonstrated that auditory stimulation can influence the cortical regions involved in balance regulation and postural control [3]. Evidence also suggests that certain auditory stimuli may reduce postural sway and improve balance performance, indicating the potential role of auditory-based interventions in postural regulation [3]. Similarly, motor coordination relies on efficient communication between the neural, sensory, and musculoskeletal systems of the body. Auditory input has been shown to contribute to motor performance through its effects on sensorimotor integration, movement timing, and motor planning [10].

Increasing evidence indicates that binaural beat stimulation may influence motor and balance-related outcomes by modulating neural oscillatory activity and sensorimotor processing [1,2]. Clinical studies have reported improvements in motor symptoms among individuals with neurological conditions following exposure to gamma-frequency auditory stimulation, suggesting a potential role for gamma oscillations in movement initiation and coordination [4]. Additionally, studies examining other binaural beat frequencies have reported improvements in hand–eye coordination, reaction accuracy, and motor performance following auditory stimulation [11].

Beyond motor outcomes, GBBs have been investigated for their effects on cognitive function. Previous studies have reported improvements in attention, focus, memory, cognitive flexibility, and executive functioning following exposure to 40 Hz binaural beat stimulation [12,13,14,15]. However, the findings remain inconsistent, as some investigations have failed to demonstrate significant effects on attention or anxiety and have reported limited neurophysiological evidence of entrainment [14,16]. Despite these mixed findings, gamma-frequency auditory stimulation remains a promising area of research because of its potential to modulate both cognitive and sensorimotor processes in the brain.

Although existing evidence suggests that auditory stimulation may influence cognitive, motor, and balance outcomes through neural entrainment mechanisms, important gaps remain. Most studies have focused on clinical populations, while relatively few have examined the potential neuromotor enhancement effects of gamma binaural beats in healthy individuals [4]. Furthermore, previous investigations have generally evaluated cognitive, balance, and coordination outcomes independently, rather than examining these domains simultaneously. To date, no study has comprehensively investigated the effects of 40 Hz GBB stimulation on postural control, motor coordination, and cognitive function in healthy Saudi individuals. Therefore, this study aimed to evaluate the effects of 40 Hz GBB auditory stimulation on postural control, motor coordination, and cognitive function in healthy adult participants.

## 2. Materials and Methods

### 2.1. Study Design

A single-arm pre–post design was used. Participants served as their own controls, allowing direct comparison of baseline and post-intervention performance, minimizing inter-individual variability. The study was approved by the Institutional Review Board of Princess Nourah bint Abdulrahman University Institutional Review Board (IRB Log Number: 25-0790).

### 2.2. Setting

The study was conducted in the Biomechanics Laboratory of the Department of Rehabilitation Sciences at Princess Nourah bint Abdulrahman University, Riyadh, Saudi Arabia. Between January and June 2026, data collection, which encompassed recruiting participants and conducting assessment sessions, was carried out.

### 2.3. Participants

**Figure 1:**
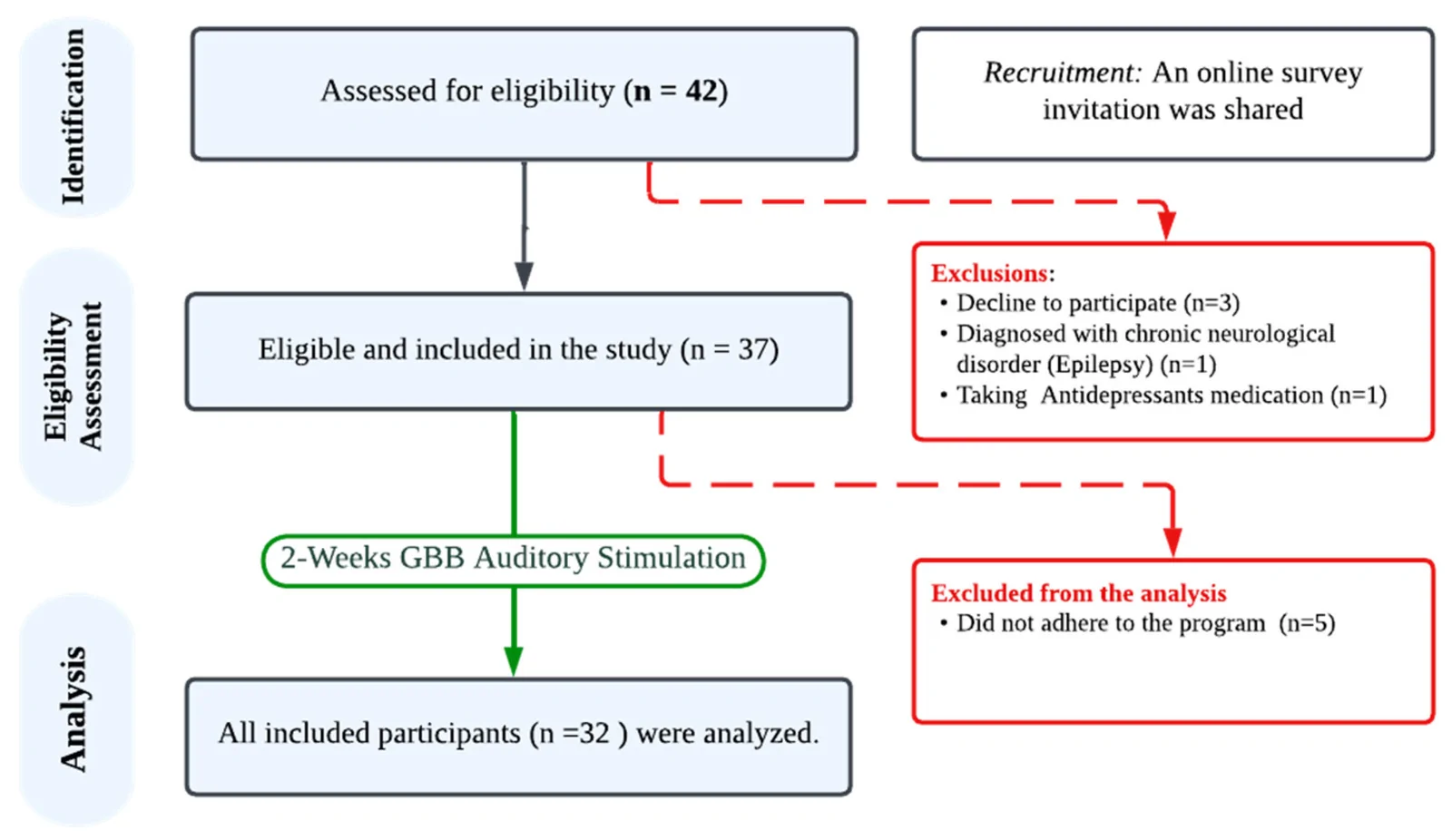
Flow diagram of the study.

Healthy adults aged 18 years and older were recruited. Participants were eligible if they were able to stand and walk independently, had normal or corrected-to-normal vision and hearing, reported no balance or coordination impairments, experienced no lower-limb pain or movement-limiting injuries, demonstrated intact cognitive function, could attend all scheduled sessions, provided written informed consent, and abstained from using medications affecting balance, stability, or alertness. Individuals were excluded if they had been diagnosed with balance or vestibular disorders, recurrent dizziness or unexplained unsteadiness, current lower-limb injuries limiting weight-bearing, medication use affecting coordination, uncorrected visual or hearing impairments, neuromuscular or orthopedic conditions restricting movement, cognitive impairment, pregnancy, a body mass index (BMI) above 40, or a history of two or more falls in the previous six months. The participant recruitment and study flow are presented in Figure 1.

### 2.4. Sample Size Calculation

The required sample size was calculated using G*Power (version 3.1) for a paired-sample (dependent) *t*-test appropriate for a pre–post experimental study design. Based on previous studies examining the effects of binaural beats on cognitive performance (e.g., Engelbregt et al. [14]), a medium effect size (Cohen’s d = 0.5) was assumed. With a statistical power of 0.80 and a two-tailed significance level of 0.05, the minimum required sample size was estimated to be 34 participants. To account for anticipated attrition, 37 participants were enrolled following eligibility screening, exceeding the a priori target.

### 2.5. Intervention

Gamma binaural beat (GBB) auditory stimulation was delivered using a standardized stereo auditory recording designed to provide separate signals to the left and right ears. The intervention used a publicly available YouTube recording described by its source as a 40 Hz GBB stimulus. The same recording was provided to all participants, who were instructed to use stereo headphones to ensure separate delivery to each ear. The recording was not generated in-house or independently subjected to acoustic or spectral verification. The recording consisted of a 440 Hz pure tone presented to the left auditory channel and a 480 Hz pure tone presented to the right auditory channel, producing a perceived binaural beat frequency of 40 Hz, corresponding to the gamma electroencephalographic (EEG) frequency range (2, 6). All participants received the same auditory recording and standardized listening instructions. They were instructed to listen to the recording for 20 min per day for two consecutive weeks while awake and alert, using stereo headphones to ensure separate delivery of the left- and right-channel signals. Participants were also instructed to complete each listening session in a quiet environment with minimal external distractions and maintain the volume at a comfortable listening level. Daily reminders were provided through the study WhatsApp channel to support adherence to the intervention. Participants used their own stereo headphones and playback devices during the home-based sessions; therefore, headphone characteristics and device output were not standardized across participants. Although participants were instructed to use a comfortable listening intensity, sound pressure level was not objectively measured or verified during home listening sessions. The research team did not independently generate or record the 440 and 480 Hz tones using dedicated laboratory software. Rather, a pre-existing auditory recording available on YouTube was used as the source of binaural beat stimulation. The recording was intended to present a 440 Hz tone to one stereo channel and a 480 Hz tone to the other channel, thereby producing a nominal interaural frequency difference of 40 Hz. Participants were provided with the same auditory recording and instructed to listen using stereo headphones to allow separate delivery of the left- and right-channel signals. The research team did not independently perform acoustic or spectral analyses.

The intervention protocol consisted of a baseline assessment session, followed by a two-week home-based auditory stimulation period and a post-intervention assessment session. During the baseline visit, the participants were assessed for static and dynamic balance, motor coordination, and cognitive function. Subsequently, the participants were instructed to listen to the 40 Hz GBB stimulus for 20 min daily over a period of two consecutive weeks using stereo headphones to ensure accurate binaural delivery. Upon completion of the intervention period, the participants returned to the laboratory for post-intervention assessments, during which all outcome measures were repeated under standardized testing conditions.

To promote adherence, the participants received daily reminders through a dedicated one-way WhatsApp channel administered by the research team. Compliance with the intervention was assessed at the end of the study using a self-reported adherence questionnaire distributed electronically through Google Forms. Participants reported the number of days on which they completed the prescribed listening sessions during the 14-day intervention period. These data were used to estimate intervention adherence and to support the interpretation of the study findings.

### 2.6. Outcome Measures

Outcome measures were conducted by physical therapists who had been trained and familiarized with the testing protocol. Prior to data collection, the student assessors received specific training on the standardized administration of the outcome measures and use of the assessment equipment. All assessments were performed according to standardized procedures to ensure consistency and accuracy across baseline and post-intervention assessments.

#### 2.6.1. Static Balance

Static balance was assessed using the ProKin Balance System, a validated force-platform–based instrument widely used for the quantitative evaluation of postural stability in both clinical and research settings [17]. Participants performed quiet standing trials under eyes-open (EO) and eyes-closed (EC) conditions to assess the contribution of visual input to postural control. The center of pressure (COP) variables included mean mediolateral displacement (COP X), mean anteroposterior displacement (COP Y), ellipse area (mm^2^), and COP perimeter (mm). In addition, Romberg-based indices were calculated to compare postural performance between the EO and EC conditions, including the EC/EO area ratio, EC/EO perimeter ratio, trunk total standard deviation, and trunk BF and ML standard deviations. Collectively, these parameters provide a comprehensive assessment of static postural stability under varying sensory conditions.

#### 2.6.2. Dynamic Balance

Dynamic balance was evaluated using the Y Balance Test (YBT), a widely accepted measure of lower extremity reach performance and neuromuscular control [18]. The participants performed maximal reach tasks in the anterior (ANT), posteromedial (PM), and posterolateral (PL) directions while maintaining a single-leg stance. Reach distances were normalized to limb length (LL) to account for anthropometric variability, and a composite score was calculated to represent overall dynamic balance performance. The composite score was calculated as follows: [(ANT + PM + PL)/(3 × LL)] × 100%. The YBT has demonstrated good-to-excellent reliability and strong inter-rater and test–retest consistency across multiple populations, supporting its use as a valid measure of dynamic postural control [19].

#### 2.6.3. Motor Coordination

Motor coordination was assessed using the Alternate Wall Toss Test, a validated measure of eye–hand coordination commonly used in motor performance research [11]. The participants stood one meter from a wall and alternately threw and caught a tennis ball using opposite hands for 30 s. One point was awarded for each successful catch, and one point was deducted for each missed or dropped catch. The final score was calculated as the total number of successful catches minus the number of errors, with higher scores indicating better coordination and reaction. Performance was categorized as excellent (>35), good (30–35), average (20–29), fair (15–19), or poor (<15).

#### 2.6.4. Cognitive Function

Cognitive function (CF) was evaluated using the PROMIS^®^ Cognitive Function Short Form 8a (Version 2.0), a reliable and validated patient-reported outcome measure designed to assess perceived cognitive abilities [20]. The instrument consists of eight items that evaluate concentration, attention, memory, and mental processing speed during the preceding seven days prior to the survey. Responses are recorded using a five-point Likert scale ranging from 1 (“Very Often”) to 5 (“Never”), with higher scores reflecting better perceived CF. The questionnaire was administered before and after the intervention to assess changes in self-reported cognitive performance following exposure to 40 Hz binaural beat stimulation.

#### 2.6.5. Participant Adherence

Adherence to the binaural beat intervention was evaluated using a self-reported compliance questionnaire administered at the end of the study. The participants reported the number of days on which they completed the prescribed daily listening sessions during the two-week intervention period. The questionnaire was distributed electronically via Google Forms, and the responses were used to estimate compliance with the intervention protocol and to facilitate the interpretation of study outcomes. Adequate adherence was defined a priori as completion of the majority of the 14 prescribed daily listening sessions. This criterion was established before data collection and was applied uniformly to all participants. Of the 37 participants enrolled, five reported during follow-up that they had not listened to the prescribed gamma binaural beat auditory stimulation. These participants were therefore considered non-adherent and were excluded from the final analysis, yielding a final analyzed sample of 32.

### 2.7. Ethical Considerations

Ethical approval was obtained from the Institutional Review Board of Princess Nourah bint Abdulrahman University (IRB Log Number: 25-0790) prior to participant recruitment and data collection. All participants received detailed information regarding the study objectives, procedures, potential risks, and participant rights before providing their written informed consent. Participation was voluntary, and the participants were free to withdraw from the study at any time without consequence. All data were collected anonymously and stored securely in accordance with institutional and international ethical guidelines to ensure confidentiality, privacy, and data integrity.

### 2.8. Statistical Analysis

All statistical analyses were performed using Stata version 27 (StataCorp LLC, College Station, TX, USA). Participant characteristics were summarized using descriptive statistics. Continuous variables, including age, height, weight, body mass index (BMI), and leg length, were presented as mean ± standard deviation (SD) with the observed range (minimum–maximum) values. Categorical variables are reported as frequencies and percentages. The normality of continuous outcome variables was assessed using the Shapiro–Wilk test and further evaluated by visual inspection of histograms and quantile–quantile (Q–Q) plots. For normally distributed variables, pre- and post-intervention differences were analyzed using paired-sample *t*-tests. In addition to statistical significance testing, effect sizes were calculated using Cohen’s d to quantify the magnitude of within-subject changes. Effect sizes were interpreted according to conventional criteria as negligible (d < 0.20), small (d = 0.20–0.49), medium (d = 0.50–0.79), or large (d ≥ 0.80). Reporting both *p*-values and effect sizes facilitated the interpretation of the statistical and practical significance of the observed changes. Outcomes were given the exploratory nature of this pilot study, no correction for multiple comparisons (e.g., Bonferroni) was applied to the family of paired-sample tests across the static postural stability, dynamic balance, motor coordination, and cognitive function outcomes; consequently, all reported *p*-values should be interpreted as nominal and hypothesis-generating rather than confirmatory, and effect sizes with 95% confidence intervals are reported alongside *p*-values to support magnitude-based interpretation. Statistical significance was established a priori at a two-tailed alpha of 0.05.

## 3. Results

### 3.1. Participant Characteristics

Thirty-seven participants were enrolled following the eligibility screening, exceeding the a priori target of 34. Five participants were subsequently excluded from the analysis for non-adherence to the intervention protocol, yielding a final sample of 32 participants. The sample was predominantly female (*n* = 31, 96.88%), with a mean age of 22.28 ± 2.43 years (range: 19–33 years). The mean body mass index (BMI) was 24.23 ± 4.41 kg/m^2^ (range: 16.95–33.74 kg/m^2^), and the mean leg length was 85.68 ± 6.69 cm (range: 58–100 cm). Right-leg dominance was reported by most participants (*n* = 27, 84.38%). Regarding sociodemographic characteristics, all participants were single, and most were students (*n* = 30, 93.75%). In terms of educational attainment, the largest proportion held a bachelor’s degree (*n* = 21, 65.62%). With respect to physical activity level, most participants were classified as having a low activity level (*n* = 23, 71.88%). The detailed demographic characteristics of all participants are presented in Table 1.

### 3.2. Dynamic Balance and Motor Coordination

Changes in dynamic balance and upper-limb coordination following 40 Hz GBB stimulation are presented in Table 2. For the Y Balance Test, the anterior (ANT) reach distance increased significantly following the intervention (baseline: 74.89 ± 18.13%LL vs. post-intervention: 95.63 ± 20.63%LL; mean difference = 20.73; *p* < 0.001; d = 1.16). Similarly, the posteromedial (PM) reach showed a statistically significant decrease after normalization to leg length (baseline: 109.01 ± 13.57%LL vs. post-intervention: 90.20 ± 16.75%LL; d = −0.98). The posterolateral (PL) reach also showed a statistically significant decrease after normalization (baseline: 87.26 ± 9.79%LL vs. post-intervention: 83.21 ± 10.99%LL; mean difference = −4.05; *p* = 0.01; d = −0.43). The composite Y Balance Test score increased significantly following the intervention (baseline: 94.09 ± 21.75% vs. post-intervention: 103.03 ± 21.31%; mean difference = 8.94; *p* < 0.001; d = 0.85).

As a sensitivity analysis, we examined the raw (non-normalized) YBT reach distances (cm) using paired-sample *t*-tests. Consistent with the interpretation above, raw reach distance increased significantly in the ANT direction (*n* = 32; pre: 63.50 ± 11.92 cm; post: 81.32 ± 15.78 cm; mean difference = 17.82 cm, 95% CI [12.29, 23.35]; t(31) = 6.57, *p* < 0.0001) and in the PL direction (*n* = 32; pre: 86.38 ± 14.38 cm; post: 90.77 ± 15.80 cm; mean difference = 4.40 cm, 95% CI [0.26, 8.53]; t(31) = 2.17, *p* = 0.038), whereas the increase in the PM direction did not reach statistical significance (*n* = 32; pre: 89.30 ± 17.16 cm; post: 90.42 ± 17.97 cm; mean difference = 1.11 cm, 95% CI [−2.55, 4.78]; t(31) = 0.62, *p* = 0.540).

It should be noted that the apparent decreases in the PM and PL reach directions occurred only in scores normalized to leg length (%LL); the corresponding raw, non-normalized reach distances increased following the intervention, consistent with the pattern observed for the ANT direction. Because %LL scores are derived by dividing the raw reach distance by leg length, this divergence reflects the influence of leg-length normalization on the resulting ratio rather than a true decline in reaching performance and represents an expected outcome once leg length is accounted for in the analysis. Therefore, we consider the composite Y Balance Test score, which combines the three reach directions normalized to leg length according to the standard YBT methodology, to be the most appropriate indicator of overall dynamic balance, consistent with the prior literature.

**Figure 2:**
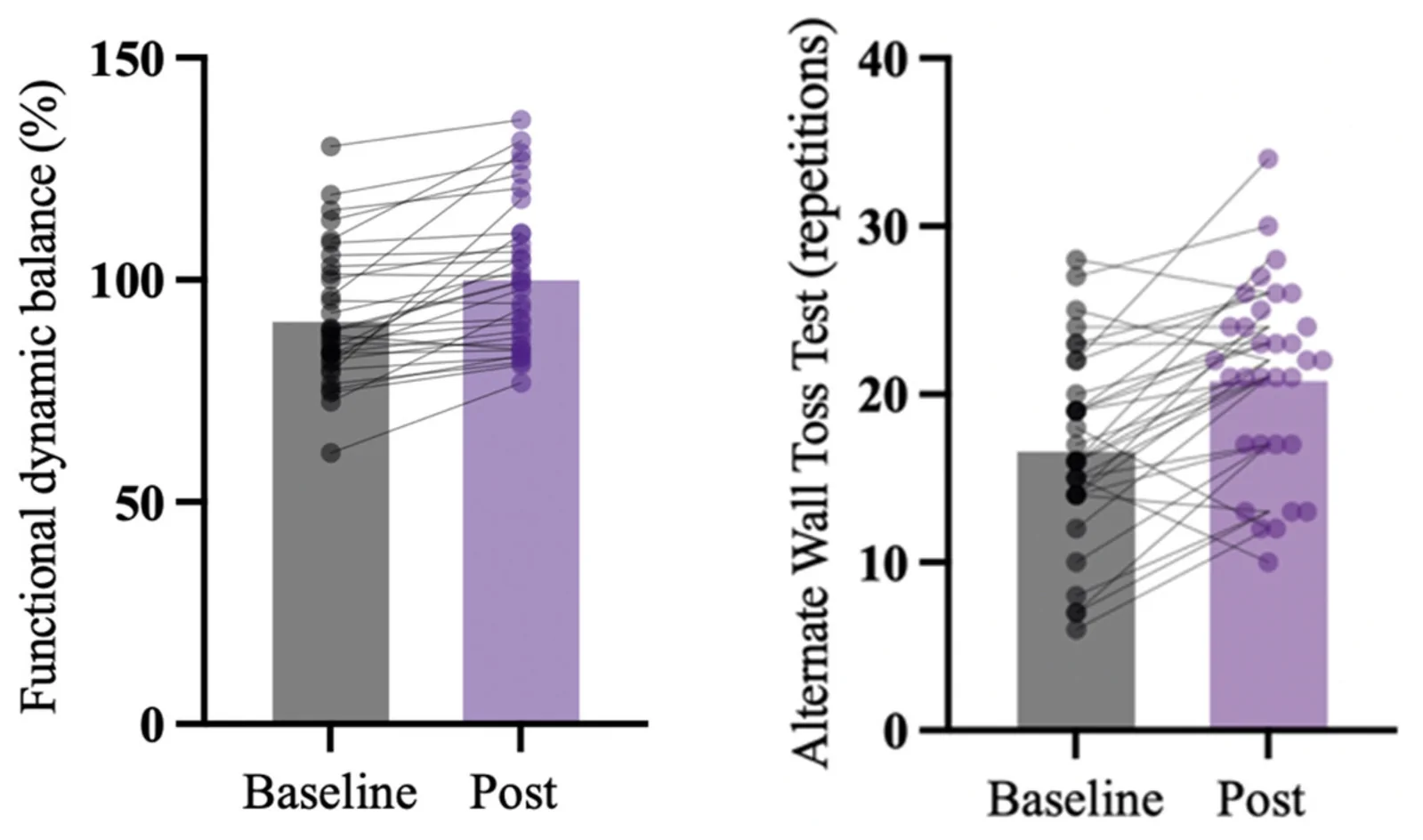
Illustrates the paired baseline- and post-intervention differences in coordination and dynamic balance outcomes following GBB auditory stimulation.

Upper limb coordination, assessed using the Alternate Wall Toss Test, improved significantly after the intervention. The mean score increased from 16.87 ± 5.70 to 21.09 ± 5.69 repetitions (mean difference = 4.21; *p* < 0.001; Cohen’s d = 0.92), representing a large effect size. The individual pre- and post-intervention changes in dynamic balance and coordination outcomes are shown in Figure 2.

### 3.3. Static Postural Stability

Changes in static postural stability are summarized in Table 2. No statistically significant differences were observed in most center of pressure (COP)-derived variables under either eyes-open (EO) or eyes-closed (EC) conditions (*p* > 0.05). The only significant change was observed in the Stability Index under EO conditions, which improved following the intervention (*p* = 0.0005; d = −0.68). Under EC conditions, none of the COP variables demonstrated statistically significant changes, with effect sizes ranging from −0.09 to 0.21. Although the COP perimeter under EO conditions approached statistical significance (*p* = 0.05; d = 0.35), it did not meet the predefined threshold.

### 3.4. Cognitive Function

The PROMIS Cognitive Function Short Form demonstrated the largest improvement (baseline: 28.56 ± 6.76 vs. post: 33.56 ± 5.09; mean difference = 5.00, 95%, *p* < 0.001, Cohen’s d = 0.96), representing a large effect size.

## 4. Discussion

This study investigated the effects of two weeks of 40 Hz GBB auditory stimulation on postural control, motor coordination, and cognitive function in healthy adults. The findings demonstrated that GBB stimulation was associated with significant improvements in dynamic balance, upper limb coordination, and self-perceived cognitive function, whereas its effects on static postural stability were limited. Improvements were primarily observed in functional tasks requiring active movement, sensorimotor integration, and coordinated motor responses, as reflected by the significant changes in dynamic balance and upper limb coordination. In contrast, static postural stability remained largely unchanged, except for an improvement in the Stability Index under eyes-open conditions, whereas no significant changes were observed under eyes-closed conditions. These findings suggest that gamma-frequency auditory stimulation may preferentially influence functional and task-oriented performance rather than quiet-standing postural control. The greater improvements observed under eyes-open conditions further indicate that the effects of GBB stimulation may be enhanced when visual input is available, highlighting the potential interaction between auditory entrainment and multisensory integration during postural regulation.

It is important to interpret this pattern of findings cautiously given the absence of a control condition. The large, statistically significant improvements were concentrated in effort- and self-report-dependent outcomes (dynamic balance, upper-limb coordination, and self-reported cognitive function), whereas the largely automatic, non-volitional measure of static postural stability showed minimal change. This cross-domain asymmetry is equally consistent with a generalized expectancy or demand-characteristic response, in which participants’ awareness of receiving an active intervention and repeated exposure to the assessment battery could plausibly improve performance on tasks sensitive to motivation and effort, while leaving largely automatic postural reflexes comparatively unaffected. Accordingly, the present results should be regarded as hypothesis-generating effect-size estimates from an exploratory pilot study rather than confirmatory evidence of a stimulation-specific mechanism, and stimulation-specific interpretations should await replication against sham or acoustically matched control conditions.

The significant improvement in PROMIS^®^ Cognitive Function scores observed in the present study is consistent with the growing evidence that gamma-frequency binaural beat stimulation may enhance cognitive performance. Wang et al. demonstrated that 40 Hz binaural beat stimulation improved working memory in healthy adults and was accompanied by neurophysiological changes, including increased EEG complexity and enhanced neural activity associated with attention, suggesting that gamma frequency stimulation may facilitate cognitive processing through neural entrainment mechanisms [9]. Similarly, Engelbregt et al. reported that exposure to 40 Hz binaural beats reduced false responses during an attention task, further supporting the potential cognitive benefits of gamma frequency auditory stimulation [14]. However, these proposed explanations remain hypothetical, as the present study did not include EEG or other neurophysiological measures to directly assess neural entrainment and cortical modulation. In contrast, Leistiko et al. found no significant improvements in attention or anxiety following gamma-frequency binaural beat stimulation [16]. The discrepancy between these findings and those of the present study may be attributable to methodological differences. Unlike the present study, which employed daily 40 Hz stimulation over a two-week period and evaluated changes in self-perceived cognitive function using the PROMIS^®^ Cognitive Function Short Form, Leistiko et al. investigated the immediate effects of a single stimulation session using the Attention Network Test, which primarily measures reaction time, error rate, and attentional network efficiency [16]. Furthermore, their remotely conducted study may have introduced greater variability in listening conditions, including headphone quality, sound intensity, environmental distractions, and background noise. Collectively, differences in intervention duration, cognitive outcome measures, participant characteristics, and experimental conditions may account for inconsistencies in the findings reported across studies.

The observed improvement in upper-limb coordination is consistent with previous evidence supporting the role of auditory stimulation in motor performance. Khan et al. demonstrated that beta-frequency binaural beats significantly enhanced eye–hand coordination in healthy young adults, highlighting the potential of auditory stimulation to facilitate coordination-related performance [11]. Although the present study employed gamma-frequency rather than beta-frequency stimulation, the significant improvement observed in the Alternate Wall Toss Test suggests that the facilitatory effects of binaural beats on motor coordination may extend across various stimulation frequencies.

The beneficial effects of auditory stimulation on motor coordination are further supported by studies investigating sensorimotor integration. Varlet et al. demonstrated that the integration of auditory and visual rhythmic cues reduced movement variability and improved synchronization compared with unimodal stimulation, emphasizing the importance of multisensory input in optimizing coordinated movements [21]. Similarly, Koh et al. reported that intra-auditory integration enhanced motor performance and multi-finger synergy during a force-production task, suggesting that auditory feedback contributes to more consistent and efficient motor output [10]. Consistent with these findings, Azizzadeh Herozi et al. showed that 30 min of alpha-frequency binaural acoustic stimulation improved digital mirror-tracing performance, with older adults committing fewer errors and younger adults completing the task more rapidly [22]. Collectively, these studies support the hypothesis that auditory stimulation enhances sensorimotor integration, movement synchronization, and visuomotor coordination. The present findings extend this evidence by demonstrating that two weeks of 40 Hz gamma binaural beat stimulation is associated with improved upper-limb coordination in healthy adults, suggesting that gamma-frequency auditory stimulation may represent a promising non-invasive approach for enhancing motor performance.

The improvements in dynamic balance observed in the present study are supported by previous evidence demonstrating the beneficial effects of auditory stimulation on balance-related performances. Chen et al. reported significant improvements in balance and functional outcomes among individuals with stroke who received binaural beat stimulation during rehabilitation, suggesting that auditory stimulation may enhance sensorimotor processes involved in postural control, even in clinical populations [3]. Similarly, Çulhaoğlu and Baylan demonstrated that binaural audio stimulation significantly improved both static and dynamic balance, as evidenced by enhanced performance on the Flamingo Test and the Y Balance Test [23]. Although these studies differed from the present investigation in terms of participant characteristics, stimulation protocols, and outcome measures, they consistently support the potential of auditory stimulation to improve functional balance and motor performance in patients with stroke.

Despite these encouraging findings, the literature is inconsistent. Gedik Toker et al. reported that neither simple auditory stimuli nor auditory–cognitive tasks produced significant improvements in balance among healthy young adults, suggesting that postural control in neurologically intact individuals may be relatively resistant to non-rhythmic auditory stimulation [24]. Likewise, Calvano et al. found that gamma-range binaural acoustic stimulation reduced resting tremor in individuals with Parkinson’s disease, although improvements in broader motor outcomes were limited [4]. These observations are consistent with the present findings, in which significant improvements were evident in dynamic balance and motor coordination, whereas changes in static postural stability were minimal. One possible explanation is that dynamic balance tasks require continuous sensorimotor integration, anticipatory postural adjustments, and coordinated motor responses, making them more responsive to cortical modulation induced by gamma-frequency auditory stimulation than to quiet standing postural tasks. Collectively, the present study extends the existing literature by demonstrating that 40 Hz GBB stimulation may preferentially enhance functional and task-oriented balance performance in healthy adults while exerting limited effects on static postural control in healthy adults.

The limited changes observed in static postural stability were among the most notable findings of the present study. Despite significant improvements in dynamic balance and upper-limb coordination, most static balance parameters remained unchanged, particularly under eyes-closed conditions. This discrepancy may reflect the distinct neural and sensory demands of static and dynamic postural control. Dynamic balance and coordination require continuous sensorimotor integration, anticipatory postural adjustments, and ongoing modulation of motor responses, which may be more responsive to cortical modulation induced by gamma-frequency auditory stimulation. In contrast, static postural control, particularly in the absence of visual input, depends more heavily on automatic vestibular, somatosensory, and subcortical mechanisms that may be less susceptible to short-term cortical entrainment. Furthermore, the effects of auditory stimulation may vary according to sensory context, task complexity, and stimulation characteristics. The relatively short intervention period may also have been insufficient to induce measurable adaptations in postural reflex pathways, while the healthy status of the participants may have resulted in a ceiling effect, limiting the potential for further improvements in static balance. Collectively, these findings suggest that 40 Hz GBB stimulation may exert domain-specific effects, with a greater influence on dynamic sensorimotor performance than on quiet standing postural control. Nevertheless, this interpretation should be considered preliminary because the present study did not directly assess the neurophysiological mechanisms underlying the observed behavioral changes.

From a clinical perspective, GBB stimulation may represent a simple, non-invasive, and cost-effective adjunct to rehabilitation and performance training. The observed improvements in dynamic balance and upper-limb coordination suggest potential applications in interventions targeting functional movement, motor learning, and sensorimotor performance, rather than static postural control alone. Previous evidence demonstrating improved coordination following auditory stimulation further supports the translational potential of this approach [11]. Nevertheless, the apparent task-specific nature of the intervention indicates that factors such as stimulation dose, intervention duration, target population, and functional objectives should be carefully considered when incorporating GBB stimulation into rehabilitation or performance-enhancement programs, as the current findings should not be interpreted as evidence of clinical efficacy.

Several limitations should be considered when interpreting the present findings, the most important of which is the lack of a control condition. Because this was a single-arm pre–post study without a sham or spectrum- and loudness-matched comparison condition, the observed changes cannot be attributed specifically to 40 Hz gamma binaural entrainment; they may equally reflect expectancy, demand characteristics, and practice effects across repeated testing. This limitation is particularly relevant given that the largest improvements occurred in effort- and self-report-dependent outcomes, while the largely automatic measure of static postural stability changed minimally, a pattern that a generalized placebo-type response could also produce. Therefore, the present findings should be interpreted as exploratory, hypothesis-generating effect-size estimates rather than confirmatory evidence of a stimulation-specific mechanism. Second, the auditory stimulus was obtained from a publicly available YouTube recording and was not independently subjected to acoustic or spectral analysis. Therefore, the exact frequency composition and spectral characteristics of the stimulus could not be independently verified, which limits the reproducibility and precise characterization of the auditory intervention. Furthermore, the PROMIS^®^ Cognitive Function Short Form assesses self-perceived cognitive functioning rather than objective cognitive performance; therefore, improvements in PROMIS scores should not be interpreted as direct evidence of enhanced cognitive performance. As the participants were aware that they were receiving the intervention, expectation and reporting effects may also have influenced their responses. The absence of participant and assessor blinding represents an additional potential source of bias. Third, the relatively small convenience sample may limit the generalizability of the results to the general population of the study. Although 37 participants were enrolled, exceeding the a priori target of 34 required for 80% power, five were subsequently excluded for non-adherence, leaving a final analyzed sample of 32. This attrition-driven reduction below the prespecified target means that the study may have been somewhat underpowered to detect smaller effects, which should be considered when interpreting the non-significant findings, particularly for static postural stability outcomes. Fourth, participants were instructed to listen to the auditory stimulation for 20 min per day over two consecutive weeks; however, the specific timing of individual listening sessions (e.g., morning, afternoon, or evening) was not objectively recorded. Furthermore, no dose–response analysis examining the relationship between auditory exposure and study outcomes was performed. In addition, the study population consisted predominantly of young female adults, which may restrict the applicability of the findings to males, older adults, and clinical populations. Although efforts were made to standardize the intervention protocol, variations in listening conditions, including headphone characteristics, environmental distractions, and individual perceptions of auditory stimuli, may have influenced the consistency of exposure. Furthermore, the two-week intervention period may not have been sufficient to induce measurable adaptations in all outcome domains, particularly in terms of static postural stability. These factors should be considered when interpreting the magnitude and consistency of observed effects.

Future studies should address these limitations of the present study. Most importantly, confirmatory trials should incorporate a sham or acoustically matched control condition to determine whether the effects reported here are specific to 40 Hz binaural entrainment or reflect expectancy or other non-specific listening-related effects. Studies should also include larger and more diverse samples, particularly individuals with neurological disorders or balance impairments, to improve external validity. Longitudinal studies incorporating extended intervention periods and follow-up assessments are warranted to determine the persistence of the observed improvements and to evaluate whether delayed effects emerge in static postural control. The inclusion of objective neurophysiological measures, such as electroencephalography, alongside functional and cognitive assessments would further enhance our understanding of the mechanisms underlying gamma-frequency auditory stimulation. Moreover, future investigations should examine the influence of stimulation parameters, including frequency, intensity, and duration, to establish optimal intervention protocols. Finally, well-designed multicenter randomized controlled trials with rigorous environmental standardization are needed to strengthen the evidence base and facilitate the translation of GBB stimulation into clinical rehabilitation and performance settings.

## 5. Conclusions

In this exploratory, single-arm pilot study, two weeks of daily 40 Hz GBB stimulation were associated with improvements in dynamic balance, upper limb coordination, and self-reported cognitive function in healthy adults, while the effects on static postural control were minimal. Because the design lacked a sham or acoustically matched control condition, these findings cannot be attributed specifically to 40 Hz binaural entrainment and may instead reflect expectancy, demand characteristics, or other nonspecific effects of the intervention. Therefore, the results should be regarded as preliminary, hypothesis-generating effect-size estimates that warrant confirmation in adequately powered, sham-controlled, matched randomized trials before stimulation-specific conclusions can be drawn.

## Figures and Tables

**Table 1:** Demographic Characteristics of Participants.

Variables		Value		
		Mean ± SD	Min	Max
Age (Years)		22.28 ± 2.43	19	33
Sex (Female), *n* (%) ^†^		31 (96.88)		
Height (cm)		160.31 ± 6.98	149	177
Weight (kg)		62.5 ± 13.34	42	93
BMI (kg/m^2^)		24.23 ± 4.41	16.95	33.74
Leg length (cm)		85.68 ± 6.69	58	100
Dominant leg, *n* (%) ^†^	Left	5(15.62)	-	-
	Right	27 (84)	-	-
Marital Status Category, *n* (%) ^†^	Single	32 (100)	-	-
	Married/Previously married (Divorced/Widowed combined)	0 (0)	-	-
Education Level Category, *n* (%) ^†^	High school/Diploma	10 (31.25)	-	-
	Bachelor’s degree	21 (65.62)	-	-
	Postgraduate degree (master’s degree/Doctorate)	1 (3.12)	-	-
Employment Status, *n* (%) ^†^	Student	30 (93.75)	-	-
	Employed	1 (3.12)	-	-
	Unemployed	1 (3.12)	-	-
Physical activity level, *n* (%) ^†^	Low activity	23 (71.88)	-	-
	Moderate activity	7 (21.88)	-	-
	High activity	2 (6.24)	-	-

Values are presented as mean ± standard deviation (SD) with range (minimum–maximum) for continuous variables. ^†^ Categorical variables are presented as frequency (*n*) and percentage (%). Physical activity was categorized as low (no exercise or 1–2 days/week), moderate (3–4 days/week), and high (≥5 days/week).

**Table 2:** Within-subject effects of gamma binaural beat auditory stimulation on postural stability, dynamic balance, coordination, and cognitive function (*n* = 32).

Variable ^‡^	Gamma Binaural Beat Auditory Stimulation		Mean Difference [95% CI]	*p*-Value	Cohen’s d ES
	Baseline N = 32 Mean ± SD	Post Intervention N = 32 Mean ± SD			
Static Postural Stability measurement					
Average COP mediolateral (ML) (mm) EO	5.93 ± 5.73	6.61 ± 5.55	0.68 [−1.66, 3.02]	0.55	0.69
Average COP anteroposterior (AP) axis (mm) EO	38.50 ± 21.67	36.56 ± 18.67	1.94 [−5.98, 9.87]	0.62	−0.08
Standard mediolateral (ML) deviation (mm) EO	2.61 ± 1.77	3.54 ± 3.56	−0.93 [−2.28, 0.41]	0.167	0.24
Standard anteroposterior (AP) deviation (mm) EO	4.90 ± 2.05	4.25 ± 2.07	0.64 [−0.32, 1.61]	0.18	−0.23
Ellipse area (mm^2^) EO	229.83 ± 186.03	246.60 ± 405.79	−16.76 [−174.53, 140.99]	0.82	0.03
Perimeter (mm) EO	145.34 ± 86.07	221.38 ± 227.71	−76.03 [−135.69, 1.62]	0.05	0.35
Average COP X (mm) EC	7.62 ± 7.65	7.62 ± 5.34	0.004 [−2.94, 2.94]	0.99	−0.0005
Average COP Y (mm) EC	−33.20 ± 25.07	−36.83 ± 34.67	3.60 [−7.92, 15.19]	0.52	0.15
Standard mediolateral (ML) deviation (mm) EC	3.96 ± 3.83	4.29 ± 3.92	−0.32 [−1.99, 1.35]	0.69	0.06
Standard anteroposterior (AP) deviation (mm) EC	7.16 ± 3.63	9.37 ± 16.29	−2.21 [−8.39, 3.97]	0.47	0.12
Ellipse area (mm^2^) EC	520.17 ± 543.15	454.88 ± 504.99	65.28 [−192.82, 323.39]	0.60	−0.09
Perimeter (mm) EC	227.41 ± 130.40	264.77 ± 195.89	−37.35 [−106.38, 31.68]	0.27	0.19
Romberg-based indices—Static Balance					
EC/EO area ratio	244.68 ± 206.89	233.54 ± 218.00	11.13 [−80.44, 102.71]	0.80	−0.04
EC/EO perimeter ratio	150.43 ± 57.26	150.65 ± 58.45	−0.21 [−21.82, 21.38]	0.98	0.003
Stability Index EO	1.56 ± 0.44	1.16 ± 0.50	0.40 [0.18, 0.61]	0.0005	−0.68
Stability Index EC	2.11 ± 0.88	1.85 ± 1.15	0.25 [−0.17, 0.69]	0.23	0.21
Functional dynamic balance					
ANT ^†^ (%LL)	74.89 ± 18.13	95.63 ± 20.63	20.73 [14.34, 27.12]	<0.001	1.16
PL ^†^ (%LL)	87.26 ± 9.79	83.21 ± 10.99	−4.04 [−7.38, −0.69]	0.01	−0.43
PM ^†^ (%LL)	109.01 ± 13.57	90.20 ± 16.75	−18.81 [−25.73, −11.89]	<0.001	−0.98
Composite score ^‡^ (%)	94.09 ± 21.75	103.03 ± 21.31	8.94 [5.16, 12.72]	<0.001	0.85
Alternate Wall Toss Test	16.87 ± 5.70	21.09 ± 5.69	4.21 [2.57, 5.86]	<0.001	0.92
PROMIS-CF	28.56 ± 6.76	33.56 ± 5.09	5.00 [3.12, 6.87]	<0.001	0.96

Abbreviations: COP, center of pressure; ML, mediolateral; AP, anteroposterior; ES, effect size; EO, eyes open; EC, eyes closed; %LL, percentage of leg length; CI, confidence interval; PROMIS-CF, Patient-Reported Outcomes Measurement Information System—Cognitive Function Short Form; ANT, anterior; PL, posterolateral; PM, posteromedial. ^†^ Normalized reach distance was calculated as: [reach distance (cm)/leg length of stance leg (cm)] × 100. ^‡^ Composite score was calculated as follows: [(ANT + PM + PL)/(3 × LL)] × 100%. Significance was set at *p* < 0.05.

## Data Availability

Data are available from the corresponding author upon reasonable request.
